# Effects of different fertilization conditions and different geographical locations on the diversity and composition of the rhizosphere microbiota of Qingke (*Hordeum vulgare* L.) plants in different growth stages

**DOI:** 10.3389/fmicb.2023.1094034

**Published:** 2023-05-04

**Authors:** Lei Wang, Handong Wang, Meijin Liu, Jinqing Xu, Haiyan Bian, Tongrui Chen, En You, Chao Deng, Youhai Wei, Tianyu Yang, Yuhu Shen

**Affiliations:** ^1^Laboratory for Research and Utilization of Qinghai Tibetan Plateau Germplasm Resources, Northwest Institute of Plateau Biology, Chinese Academy of Sciences, Xining, China; ^2^Key Laboratory of Adaptation and Evolution of Plateau Biota, Northwest Institute of Plateau Biology, Chinese Academy of Sciences, Xining, China; ^3^Qinghai Provincial Key Laboratory of Crop Molecular Breeding, Northwest Institute of Plateau Biology, Chinese Academy of Sciences, Xining, China; ^4^Gannan Institute of Agricultural Sciences, Hezuo, China; ^5^University of Chinese Academy of Sciences, Beijing, China; ^6^Academy of Agriculture and Forestry Science, Qinghai University, Xining, China; ^7^Crop Research Institute, Gansu Academy of Agricultural Sciences, Lanzhou, China; ^8^Innovation Academy for Seed Design, Chinese Academy of Sciences, Xining, China

**Keywords:** Qingke, rhizosphere microbiota, fertilization condition, geographical location, diversity

## Abstract

**Introduction:**

The excessive use of chemical fertilizer causes increasing environmental and food security crisis. Organic fertilizer improves physical and biological activities of soil. Rhizosphere microbiota, which consist of highly diverse microorganisms, play an important role in soil quality. However, there is limited information about the effects of different fertilization conditions on the growth of Qingke plants and composition of the rhizosphere microbiota of the plants.

**Methods:**

In this study, we characterized the rhizosphere microbiota of Qingke plants grown in three main Qingke-producing areas (Tibet, Qinghai, and Gansu). In each of the three areas, seven different fertilization conditions (m1–m7, m1: Unfertilized; m2: Farmer Practice; m3: 75% Farmer Practice; m4: 75% Farmer Practice +25% Organic manure; m5: 50% Farmer Practice; m6: 50% Farmer Practice +50% Organic manure; m7: 100% Organic manure) were applied. The growth and yields of the Qingke plants were also compared under the seven fertilization conditions.

**Results:**

There were significant differences in alpha diversity indices among the three areas. In each area, differences in fertilization conditions and differences in the growth stages of Qingke plants resulted in differences in the beta diversity of the rhizosphere microbiota. Meanwhile, in each area, fertilization conditions, soil depths, and the growth stages of Qingke plants significantly affected the relative abundance of the top 10 phyla and the top 20 bacterial genera. For most of microbial pairs established through network analysis, the significance of their correlations in each of the microbial co-occurrence networks of the three experimental sites was different. Moreover, in each of the three networks, there were significant differences in relative abundance and genera among most nodes (i.e., the genera *Pseudonocardia*, *Skermanella*, *Pseudonocardia*, *Skermanella*, *Aridibacter,* and *Illumatobacter*). The soil chemical properties (i.e., TN, TP, SOM, AN, AK, CEC, Ca, and K) were positively or negatively correlated with the relative abundance of the top 30 genera derived from the three main Qingke-producing areas (*p* < 0.05). Fertilization conditions markedly influenced the height of a Qingke plant, the number of spikes in a Qingke plant, the number of kernels in a spike, and the fresh weight of a Qingke plant. Considering the yield, the most effective fertilization conditions for Qingke is combining application 50% chemical fertilizer and 50% organic manure.

**Conclusion:**

The results of the present study can provide theoretical basis for practice of reducing the use of chemical fertilizer in agriculture.

## Introduction

Chemical fertilizers are compounds which contain high concentrations of nutrients required during plant growth (Pahalvi et al., 2021). Although the employment of chemical fertilizers in agriculture plays an important role in increasing soil fertility and crop productivity (Savci, 2012; Liang et al., 2021), their negative impacts cannot be ignored particularly when sustainable agriculture is the global target. Excessive and continuous use of chemical fertilizers causes soil organic matter degradation, soil acidity, and environmental pollution, which also posed a threat to the food security (Savci, 2012; Pahalvi et al., 2021). Moreover, the overuse of chemical fertilizers could result in an imbalance in the N–P–K ratio and a decrease in the diversity of soil microorganisms (Li et al., 2022).

In recent years, the use of organic fertilizer increases obviously. Studies showed that organic fertilizer improves physical and biological activities of soil. But the organic fertilizer has comparatively low nutrient content, larger quantity is required for plant growth. Hence, the combined application of chemical fertilizers and organic fertilizer is sustainable and cost-effective strategy of soil fertility (Xiao et al., 2017; Bhatt et al., 2019). In China, the application of chemical fertilizers increases the yields of grain crops by 30%–60% (Adesemoye et al., 2009). Although the utilization of chemical fertilizers in China is less than that in European and American countries, the overuse of chemical fertilizers not only hardens the soil and reduces soil fertility, but also pollutes the environment. Therefore, China has encouraged reductions in the use of chemical fertilizers and the use of pesticides, aiming to develop green and sustainable modern agriculture and promote farmers’ income (Mäder et al., 2002; Fan et al., 2019).

Soil, which is an integral part of terrestrial ecosystems, is a key reservoir of global biodiversity. Soil microorganisms maintain the fertility and productivity of soil by decomposing organic matter and improving nutrient cycling in soil (Delgado-Baquerizo et al., 2020; D’hondt et al., 2021; Zheng et al., 2021). In addition, the microorganisms can regulate the activity of soil enzymes and improve the physical and chemical properties of soil, reducing nitrogen loss through ammonia volatilization and decreasing the fixation of phosphorus and potassium in soil. Thus, soil microorganisms can improve the fertility of soil and availability of nutrients to plants (Hiruma et al., 2016; Kaur et al., 2022; Li et al., 2022).

The presence of numerous beneficial microorganisms in the root zones of plants not only promotes plant growth, but also improves plant resistance to abiotic stresses and diseases (Qi et al., 2022). The microorganisms are influenced by the physical and chemical properties of soil, but at the same time they can influence the quality of soil. Studies have proven that soil microorganisms effectively maintain soil health and improve soil fertility (Chaparro et al., 2012; Ellouze et al., 2013). Maintaining the compositional diversity and functional diversity of the rhizosphere microbial community is essential to achieving sustainable ecosystem functions (Maestre et al., 2021; Nayfach et al., 2021).

The Qinghai-Tibet Plateau has a unique geographical location, unique soil types, and harsh environmental conditions (low oxygen levels, high levels of ultraviolet radiation, and low temperatures). Qingke (*Hordeum vulgare* L. var. *nudum* Hook. f.), which is also known as hull-less barley, belongs to the genus Hordeum in the family Poaceae. It is the most commonly cultivated crop in the Qinghai-Tibet Plateau (Badr et al., 2000). Qingke contains low amylose, high β-glucan, flavonoids, and other nutritional components. Qingke and its products are widely used as food, animal feed, and malt (Zeng et al., 2018). As the demand for Qingke continues to increase, the area allocated for Qingke production expands, and there is a need for a high yield of Qingke (Gong et al., 2014). However, the traditional cultivation of Qingke is prone to the overuse of chemical fertilizers. Qingke plays an important role in the sustainable management and maintenance of soil in highland agriculture (Zeng et al., 2018; Guan et al., 2022).

However, the effects of reducing the application of fertilizers on the diversity and structure of the rhizosphere microbiota of Qingke plants grown in different plateaus are not yet clear. Therefore, this study aims to characterize the rhizosphere microbiota of Qingke plants grown under different fertilization conditions in three main Qingke-producing areas (Tibet, Qinghai, and Gansu). The composition and diversity of the rhizosphere microbiota were investigated by high-throughput 16S rRNA gene sequencing. The knowledge about the diversity and composition of the rhizosphere microbiota of Qingke plants and the understanding of the relationships between the diversity and composition of the microbiota and the availability of nutrients in soil will serve as a basis for the sustainable production of Qingke in the Qinghai-Tibet Plateau.

## Materials and methods

### Experimental sites and experimental design

This study began in 2020 in three experimental sites: Haibei Agricultural and Animal Husbandry Sciences Institute, which is located in Qinghai Province; Gannan Institute of Agricultural Sciences, which is located in Gansu Province; and Bailang Agricultural Science Institute, which is located in Tibet.

Crop rotation and seven fertilization conditions were applied in each of the three experimental sites. The fertilizers used in this study were urea (46% N), diammonium hydrogen phosphate (18% N and 46% P_2_O_5_), and organic manure [48.6% organic matter (OM)]. Seven fertilizer treatments were set up and three replicates were set for every treatment at three sites. The amounts of fertilizers used for every treatment are shown in Supplementary Table 1. The agronomic characteristics of Qingke plants in different growth stages (the seedling stage, flowering stage, and mature stage) were observed. The panicle number per unit area, grain number per panicle, 1,000-grain weight, yield, and plant height were measured by sampling mature stage of Qingke plants.

### Soil sampling

In each of the three experimental sites (HB, BL, and HZ), soil samples had been collected as the original soil from the depths of 0–20, 20–40, 40–60, 60–80, and 80–100 cm using an auger with an internal diameter of 5 cm before sowing Qingke seeds. In each site, from each soil depth, three soil samples were collected. Samples were labeled as “HB-1st-(depth),” “BL-1st-(depth),” and “HZ-1st-(depth).” The soil samples were stored at 4°C before further experiments.

In the three-experiment site, the rhizosphere soil samples were collected at three Qingke development stages (i.e., seeding, flowering, and mature stages). For each stage at one site, rhizosphere soil samples of Qingke plants under seven fertilizer treatment were collected, and three replicates samples were collected for each treatment. Samples were also marked by sites and Qingke development stages [i.e., seeding stage: “HB-2nd (m1–m7),” “BL-2nd (m1–m7),” and “HZ-2nd (m1–m7)”; flowering stage: “HB-3rd (m1–m7),” “BL-3rd (m1–m7),” and “HZ-3rd (m1–m7)”; and mature stage: “HB-4th (m1–m7),” “BL-4th (m1–m7),” and “HZ-4th (m1–m7)].”

### Analysis of chemical properties of soil samples

Twelve soil chemical variables were measured, namely PH, total nitrogen (TN), total phosphorus (TP), total potassium (TK), available nitrogen (AN), available phosphorus (AP), available potassium (AK), soil organic matter (SOM), exchangeable Ca^2+^ (Ca), exchangeable Mg^2+^ (Mg), exchangeable K^+^ (K), exchangeable Na^+^ (Na), and cation exchange capacity (CEC). PH of soil extract samples was determined by potentiometric method. Soil TN was measured using Automatic Kjeldahl Nitrogen Analyzer (Sweden Foss Tecator Co.). Molybdenum antimony resistance colorimetric method was used to determine soil TP. Soil TK and AK were measured by flame photometric method. AN was determined using alkali solution diffusion method. AP was measured with sodium bicarbonate methods (0.5 M NaHCO3). Dichromate oxidation method was performed to evaluate soil SOM. Exchangeable K, Na, Ca, and Mg were extracted from soil by shaking in 1 mol/L ammonium acetate. Extracted elements were analyzed with flame atomic absorption spectrometry. Ammonium acetate method was used to determine CEC.

### 16S rRNA gene sequencing

Microbial DNA was extracted from rhizosphere samples using the FastDNA Spin Kit for Soil (MP Biomedicals, United States) and a FastPrep-24 instrument (MP Biomedicals, United States) according to the manufacturer’s instructions. The V3–V4 region of the 16S rRNA gene was amplified by an eight-base-pair barcode and the primer pair 338F (5′-ACTCCTACGGGAGGCAGCAG-3′) and 806R (5′-GGACTACHVGGGTWTCTAAT-3′). The PCR product was purified by the QIAquick Gel Extraction Kit (Qiagen, Germany) and sequenced on the Illumina MiSeq platform with the MiSeq Regen Kit v3 (Illumina, United States; the PE300 mode).

### Analysis of sequence data

Paired-end reads were merged using USEARCH v11.066721 (Edgar and Flyvbjerg, 2015). The reads were demultiplexed, and the sequences of the barcode and primer pair were cut with Cutadapt v2.11 (Kechin et al., 2017). The sequences were subjected to quality filtering with a maxEE cutoff of 1.0 and dereplicated, and unique sequences were clustered into ZOTUs using USEARCH v11.0667. The representative sequences were classified by the SINTAX algorithm using the Ribosomal Database Project (RDP) 16S v16 training set with a cutoff of 0.8. In addition, in QIIME2 v2018.10 (Bolyen et al., 2019), a phylogenetic tree was constructed by inserting the representative sequences into the Greengenes 13_8 99% tree using the SEPP algorithm (Janssen et al., 2018).

The alpha diversity and beta diversity of the rhizosphere microbiota of Qingke plants were analyzed by rarifying the ZOTU table to the minimum number of reads (i.e., 25,135 reads); estimating observed ZOTU richness, Faith’s PD, and Shannon index using QIIME2 v2018.10; and calculating JSD using the R package phyloseq v1.32.0 (McMurdie and Holmes, 2013). The correlations between the structures of the rhizosphere microbial communities of Qingke plants and macronutrients were evaluated with the envfit function of the R package vegan v2.5–6 (Oksanen et al., 2013). On the other hand, the PERMANOVA of the effects of different geographical locations and different fertilization conditions on the rhizosphere microbiota of Qingke plants was performed using the adonis function of the vegan package.

Spearman’s correlation analysis was performed on microbial genera to construct microbial co-occurrence networks. The correlation analysis was performed using Cytoscape v3.5.1 and the igraph package in R (Cline et al., 2007). The microbial genera, whose average relative abundance in soil is above 0.1%, were used to construct microbial co-occurrence networks by Spearman’s correlation analysis. Microbial pairs with significant correlations (*p-*value < 0.01) were used to establish microbial co-occurrence networks.

### Statistical analysis

The significance levels of multiple comparisons were adjusted using the Benjamini–Hochberg procedure. Adjusted *p*-values smaller than 0.05 were considered statistically significant. One-way ANOVA were corrected using the Benjamini-Hochberg false discovery rate (FDR) algorithm with a significance level of 0.05 (*p.*adj value). *p.*adj smaller than 0.05 were considered as statistically significant unless otherwise specified.

## Results

### Sequence data and diversity of the rhizosphere microbiota of Qingke plants

Two hundred and thirty-four soil samples were collected from three experimental sites located in three main Qingke-producing areas (Supplementary Figure 1). The experimental sites were Haibei Agricultural and Animal Husbandry Sciences Institute (HB), which is located in Qinghai Province; Gannan Institute of Agricultural Sciences (HZ), which is located in Gansu Province; and Bailang Agricultural Science Institute (BL), which is located in Tibet Autonomous Region. The effects of different fertilization conditions (m1–m7, m1: Unfertilized; m2: Farmer Practice; m3: 75% Farmer Practice; m4: 75% Farmer Practice +25% Organic manure; m5: 50% Farmer Practice; m6: 50% Farmer Practice +50% Organic manure; m7: 100% Organic manure) and different growth stages of Qingke plants (the seedling, flowering, and mature stages; Supplementary Table 1) on the rhizosphere microbiota of the plants were investigated.

The rhizosphere microbiota of Qingke plants were profiled by sequencing the V3–V4 region of the 16S rRNA gene. For each rhizosphere sample, the median number of reads was 43,255 (the number of reads ranged from 25,135 to 89,620). The reads were clustered into 37,785 zero-radius operational taxonomic units (ZOTUs), and 60.11% of these ZOTUs (accounting for 87.72% of the total reads) were assigned to 431 genera belonging to 24 phyla. After the normalization of sequencing depth to the minimum number (i.e., 25,135 reads), diversity analysis was performed to investigate the effects of different geographical locations and different fertilization conditions on the alpha diversity and beta diversity of the rhizosphere microbiota of Qingke plants.

**Figure 1:**
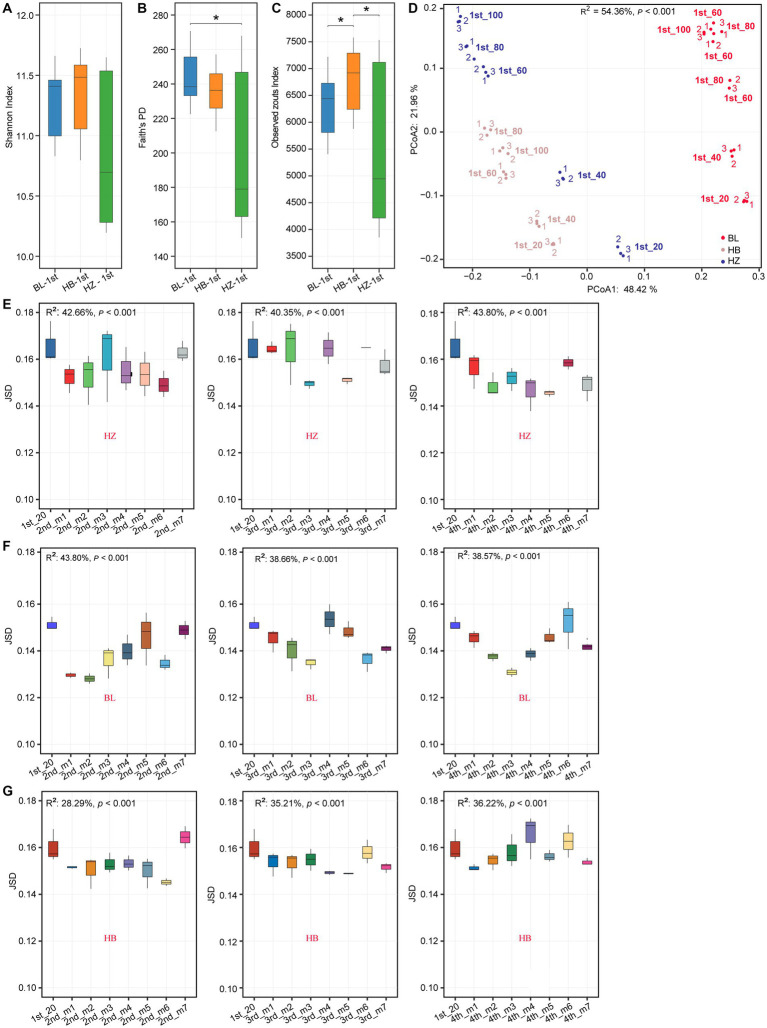
Rhizosphere microbiota diversity changes by different geographical locations and fertilization conditions. **(A–C)** Shannon index, Faith’s PD and Observed Zotus of original soil microbiota at the three experimental sites. ^*^p.adj < 0.05, ^**^p.adj < 0.01, ^***^p.adj < 0.001, Mann–Whitney test. **(D)** PCoA plot based on JSD of the original soil microbial community at different soil depths of the three experimental sites. Corresponding R^2^ and *p* values for PERMANOVA test were shown. 20, 40, 60, 80, 100: the depths of 0–20, 20–40, 40–60, 60–80, and 80–100 cm of the soil samples. Samples were labeled as “1st-(depth).” Red, blue, and coffee colors represent the experimental site of HB, HZ, BL, respectively. In each site, from each soil depth, three soil samples were collected, and which were labeled as “1,” “2,”“3,” respectively. **(E–G)** Inter-group JSD of the rhizosphere microbial community of Qingke plants at the different fertilization conditions and different growth stages at the three experimental sites. The center line of boxplot represents the median, box limits represent upper and lower quartiles and whiskers represent 1.5× interquartile range. 1st: before sowing Qingke seeds. 2nd: the seeding stage of the Qingke plants. 3rd: the flowering stage of the Qingke plants. 4th: the mature stage of the Qingke plants. m1: Unfertilized; m2: Farmer Practice; m3: 75% Farmer Practice; m4: 75% Farmer Practice +25% Organic manure; m5: 50% Farmer Practice; m6: 50% Farmer Practice +50% Organic manure; m7: 100% Organic manure. HB: The experimental site was Haibei Agricultural and Animal Husbandry Sciences Institute in Qinghai Province, HZ: The experimental site was Gannan Institute of Agricultural Sciences in Gansu Province; BL: the experimental site was Bailang Agricultural Science Institute in Tibet Autonomous Region.

Diversity differing between the tree experimental sites were identified using Wilcox test. There were significant differences in alpha diversity indices (Shannon index, Faith’s phylogenetic diversity (Faith’s PD), and observed ZOTU richness) among original soil of the three experimental sites (BL, HB, and HZ; Figures 1A–C). Among the three experimental sites, BL had the highest Faith’s PD. On the other hand, the observed ZOTU richness of HB was significantly higher (*p*-value < 0.05) than that of BL and HZ. In addition to the significant differences in alpha diversity indices among the three sites, there were significant differences in the structure of the rhizosphere microbial community (beta diversity) among the sites (Figure 1D; permutational multivariate analysis of variance (PERMANOVA), *p-*value < 0.001). The structure of the rhizosphere microbial community was estimated by Jenson–Shannon divergence (JSD), and normalized sequence data were used in the calculation of JSD. The results of principal coordinate analysis (PCoA) showed that the first two axes explained 54.36% of the total variation: PCo1 and PCo2 explained 48.42% and 21.96% of the total variation, respectively.

Meanwhile, the rhizosphere microbial community structure was evaluated too. In the present study, as evaluated by the envfit function, the structures of the soil microbial communities (i.e., beta diversity, which was estimated by JSD) of Qingke plants significantly correlated (adjusted value of *p* < 0.05) with 10 of the soil chemical properties (Supplementary Figure 2). In addition, the structures of the soil microbial communities were affected by fertilization conditions and the growth stages of Qingke plants (the seedling stage, flowering stage, and mature stage; Figures 1E–G). At a constant soil depth (20 cm), in each of the three experimental sites, due to different fertilization conditions and different growth stages of Qingke plants, there were differences in the structures of the rhizosphere microbial communities of Qingke plants.

### Effects of different fertilization conditions and different growth stages of Qingke plants on the composition of the rhizosphere microbiota of the plants

**Figure 2:**
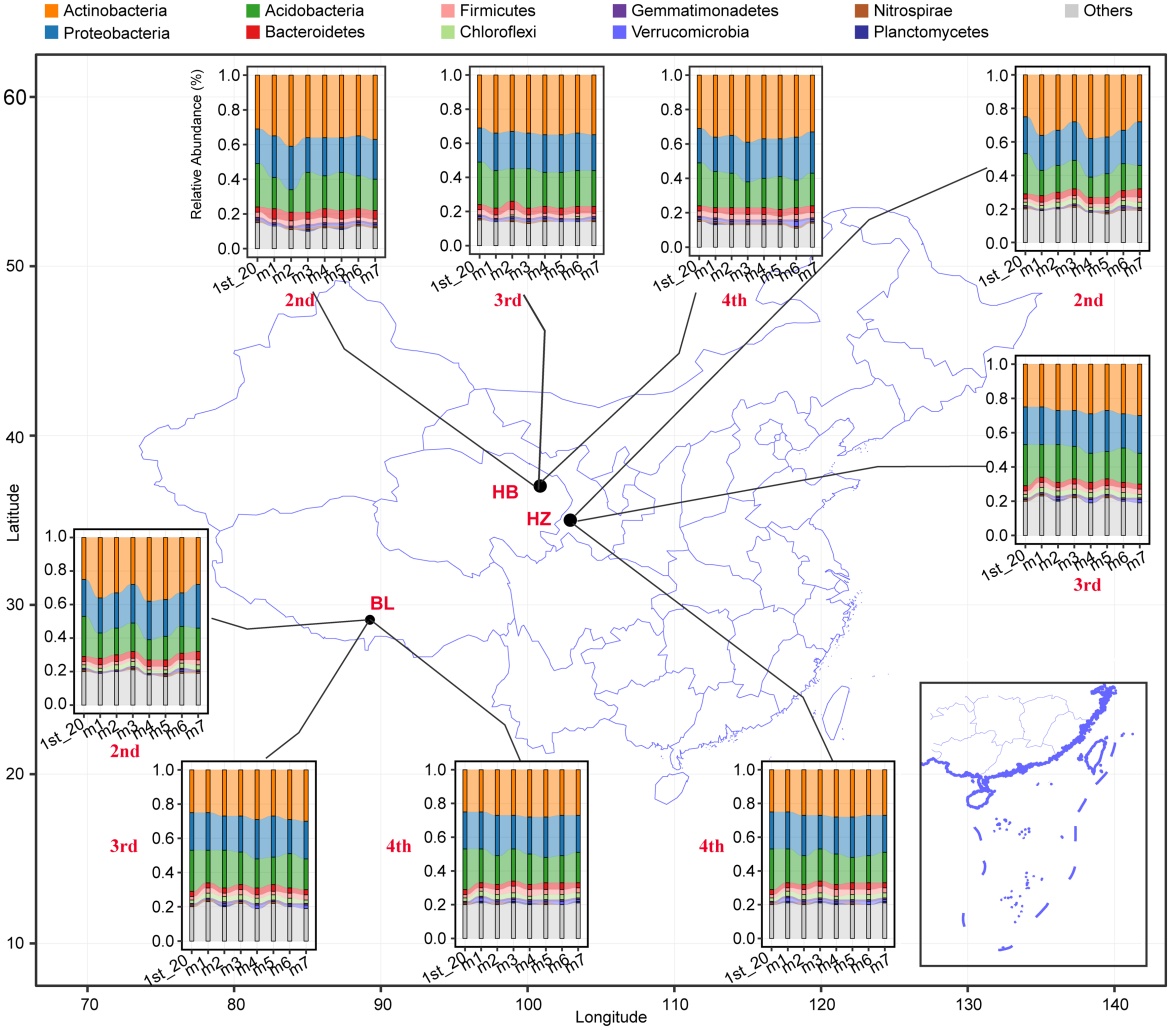
The top 10 phyla composition distribution of the rhizosphere microbiota at the different fertilization conditions and different growth stages of Qingke plants. 1st: before sowing Qingke seeds, 2nd: the seeding stage of the Qingke plants. 3rd: the flowering stage of the Qingke plants. 4th: the mature stage of the Qingke plants. m1: Unfertilized; m2: Farmer Practice; m3: 75% Farmer Practice; m4: 75% Farmer Practice +25% Organic manure; m5: 50% Farmer Practice; m6: 50% Farmer Practice +50% Organic manure; m7: 100% Organic manure. HB: the experimental site was Haibei Agricultural and Animal Husbandry Sciences Institute in Qinghai Province, HZ: the experimental site was Gannan Institute of Agricultural Sciences in Gansu Province; BL: the experimental site was Bailang Agricultural Science Institute in Tibet Autonomous Region.

The effects of different fertilization conditions and different growth stages of Qingke plants on the phylum-level composition of the rhizosphere microbiota of the plants were analyzed (Figure 2). Actinobacteria, Proteobacteria, Acidobacteria, Bacteroidetes, Firmicutes, Chloroflexi, Gemmatimonadetes, Verrucomicrobia, Nitrospira, and Planctomycetes were the 10 abundant bacterial phyla in all rhizosphere samples. In each of the three experimental sites, fertilization conditions and the growth stages of Qingke plants significantly affected the relative abundance of the top 10 phyla (Supplementary Table 2). As shown in Figure 2, across the three experimental sites, the relative abundance of Actinobacteria, Proteobacteria, and Acidobacteria is higher than that of the other seven phyla. In HB, the seven different fertilization conditions and three growth stages of Qingke plants significantly affect the relative abundance of Bacteroidetes, Firmicutes, and Gemmatimonadetes. In HZ, the seven fertilization conditions and three growth stages of Qingke plants significantly affect the relative abundance of Bacteroidetes. In BL, the relative abundance of Bacteroidetes, Firmicutes, Chloroflexi, and Gemmatimonadetes is significantly affected by the seven fertilization conditions and four growth stages of Qingke plants. In summary, fertilization conditions and the growth stages of Qingke plants are factors that affect the composition of the rhizosphere microbiota of Qingke plants.

**Figure 3:**
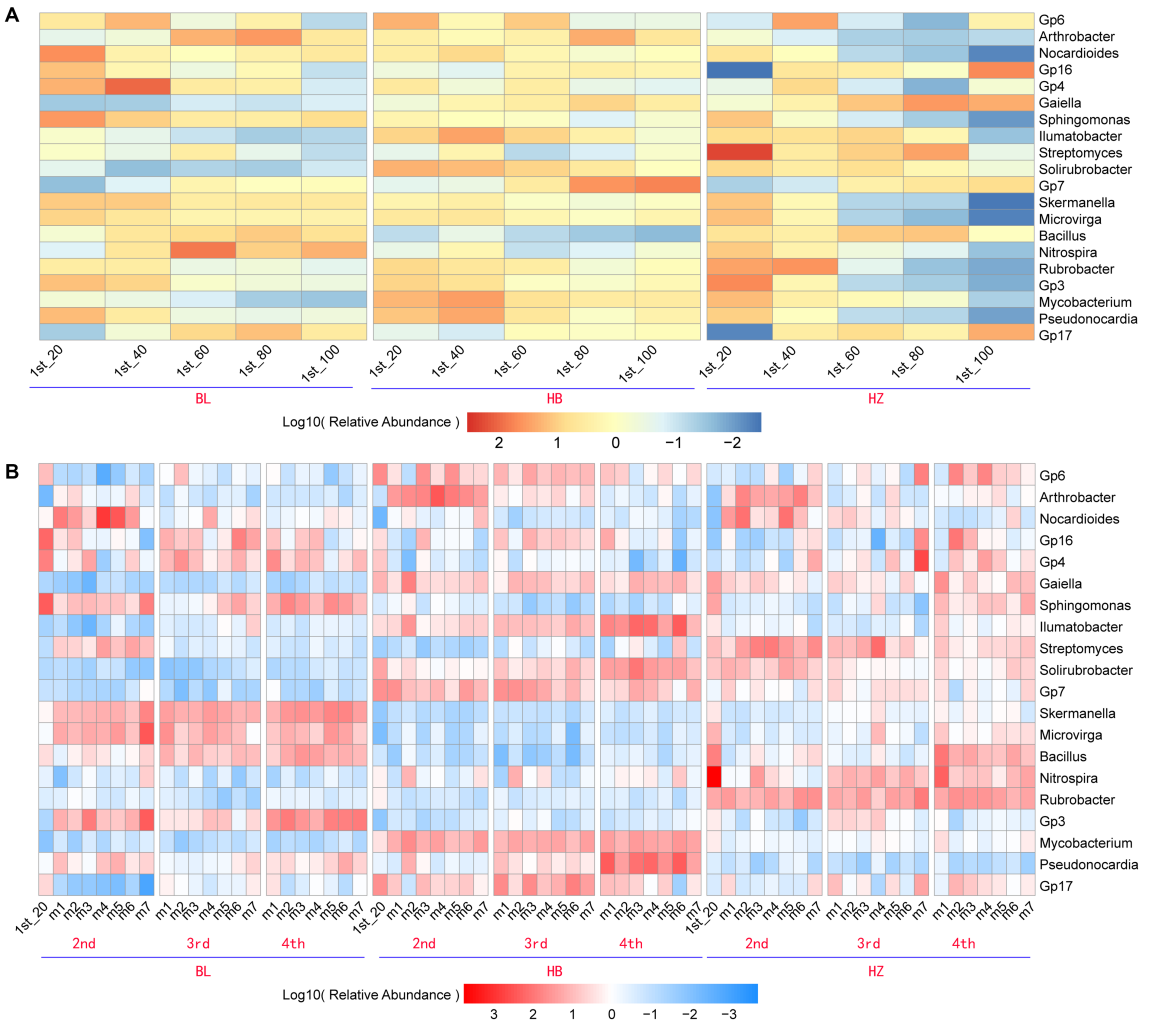
The core genera characteristics of the rhizosphere microbiota of Qingke plants at the different fertilization conditions and different growth stages. **(A)** The relative abundance of core genera of the original soil microbiota between the different depths at the three experiments sites. **(B)** The relative abundance of core genera of the rhizosphere microbiota between the different fertilization conditions and different growth stages of Qingke plants at the three experimental sites. The core genera were selected with an average relative abundance > 0.1% in all soil samples. 1st: before sowing Qingke seeds. 2nd: the seeding stage of the Qingke plants. 3rd: the flowering stage of the Qingke plants. 4th: the mature stage of the Qingke plants. m1: Unfertilized; m2: Farmer Practice; m3: 75% Farmer Practice; m4: 75% Farmer Practice +25% Organic manure; m5: 50% Farmer Practice; m6: 50% Farmer Practice +50% Organic manure; m7: 100% Organic manure. HB: the experimental site was Haibei Agricultural and Animal Husbandry Sciences Institute in Qinghai Province, HZ: the experimental site was Gannan Institute of Agricultural Sciences in Gansu Province; BL: the experimental site was Bailang Agricultural Science Institute in Tibet Autonomous Region.

Due to the significant effects of fertilization conditions and the growth stages of Qingke plants on the phylum-level composition of the rhizosphere microbiota of the plants, the effects of fertilization conditions, soil depths, and the growth stages of Qingke plants on the genus-level composition of the rhizosphere microbiota were investigated. The top 20 bacterial genera (i.e., the genera were selected with an average relative abundance > 0.1% in all soil samples) in all original soil samples were Gp6, *Arthrobacter*, *Nocardioides*, Gp16, Gp4, *Gaiella*, *Sphingomonas*, *Illumatobacter*, *Streptomyces*, *Solirubrobacter*, Gp7, *Skermanella*, *Microvirga*, *Bacillus*, *Nitrospira*, *Rubrobacter*, Gp3, *Mycobacterium*, *Pseudonocardia*, and Gp17 (Figure 3A). In each of the three experimental sites, soil depths and fertilization conditions significantly affected the relative abundance of the top 20 genera (Supplementary Table 3). As shown in Figure 3A, in each of the three experimental sites, the five depths of soil (20, 40, 60, 80, and 100 cm) influence the relative abundance of the top 20 genera. For example, in BL, the genus Gp16 is enriched at the soil depth of 40 cm, and the genus Bacillus is enriched at the soil depth of 60 cm. On the other hand, in HZ, the genus Streptomyces is enriched at the soil depth of 20 cm.

In each of the three experimental sites, fertilization conditions and the growth stages of Qingke plants significantly affected the relative abundance of the top 20 genera (i.e., the genera were selected with an average relative abundance > 0.1% in all soil samples, Figure 3B). As shown in Figure 3B, the seven fertilization conditions (m1–m7) and three growth stages of Qingke plants (i.e., the seedling, flowering, and mature stages) affect the relative abundance of the top 20 genera in the three experimental sites (Supplementary Table 4). In general, the relative abundance of *Nocardioides*, *Sphingomonas*, *Skermanella*, *Bacillus*, *Microvirga*, and Gp3 in BL is significantly higher than that of the six genera in HB and HZ. The genera *Nocardioides* is enriched in Qingke plants in the seedling stage, while the genera *Sphingomonas* is enriched in Qingke plants in the mature stage at BL. On the other hand, the relative abundance of Gp6, *Arthrobacter*, *Illumatobacter*, *Solirubrobacter*, *Mycobacterium*, *Pseudonocardia*, and Gp17 in HB is significantly higher than that of the seven genera in HZ and BL. The genera *Arthrobacter* is enriched in Qingke plants in the seedling stage, while the genera *Illumatobacter* and *Pseudonocardia* are enriched in Qingke plants in the mature stage at HB. The relative abundance of Streptomyces, *Nitrospira*, and *Rubrobacter* in HZ is significantly higher than that of the three genera in HB and BL.

### Microbial co-occurrence networks of the three experimental sites

**Figure 4:**
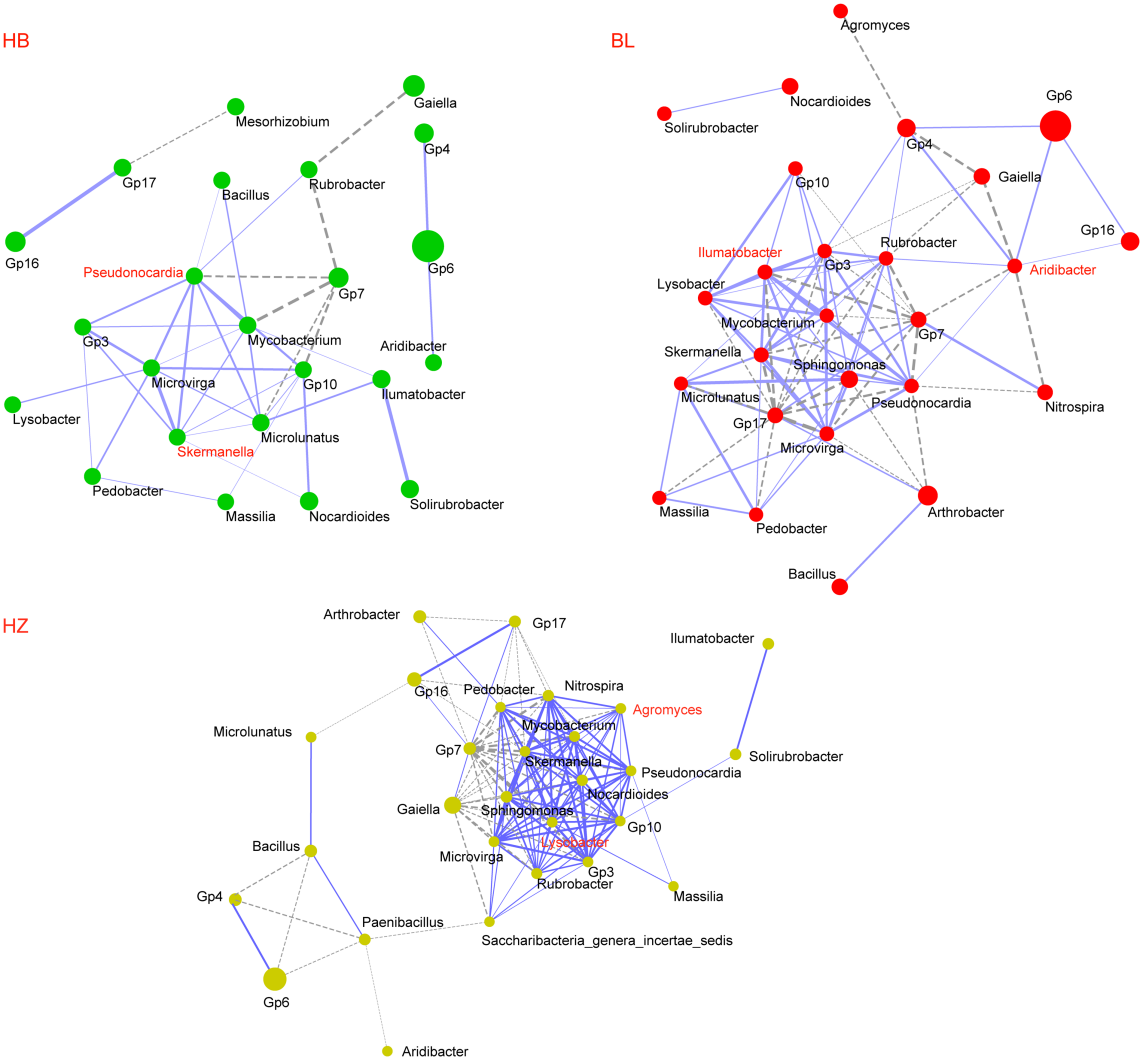
Co-occurrence network of core genera of the rhizosphere microbiota of the three experiments sites. The core genera were selected with an average relative abundance > 0.1% in all soil samples. Network correlations with r > 0.5 and *p* < 0.05 were shown. Each node represents a genus; the size of the node is proportional to the mean relative abundance of the corresponding genus; the solid and dotted lines represent positive and negative correlations, respectively. HB: the experimental site was Haibei Agricultural and Animal Husbandry Sciences Institute in Qinghai Province, HZ: the experimental site was Gannan Institute of Agricultural Sciences in Gansu Province; BL: the experimental site was Bailang Agricultural Science Institute in Tibet Autonomous Region.

The three experimental sites (HB, BL, and HZ) differed in the alpha diversity and beta diversity of the rhizosphere microbiota of Qingke plants; the possibility that the three sites differ in interactions between members of the rhizosphere microbiota was investigated. As shown in Figure 4, the microbial co-occurrence networks of the three experimental sites are distinctly different. The microbial co-occurrence network of HZ (28 nodes and 135 edges) is larger than those of BL (26 nodes and 94 edges) and HB (24 nodes and 42 edges). Nineteen nodes are shared by the three microbial co-occurrence networks, but only 22 edges are shared by them; for the same microbial pairs, the significance of their correlations in each of the three microbial co-occurrence networks is different. Moreover, the three microbial co-occurrence networks have different hubs (i.e., highly connected nodes).

**Figure 5:**
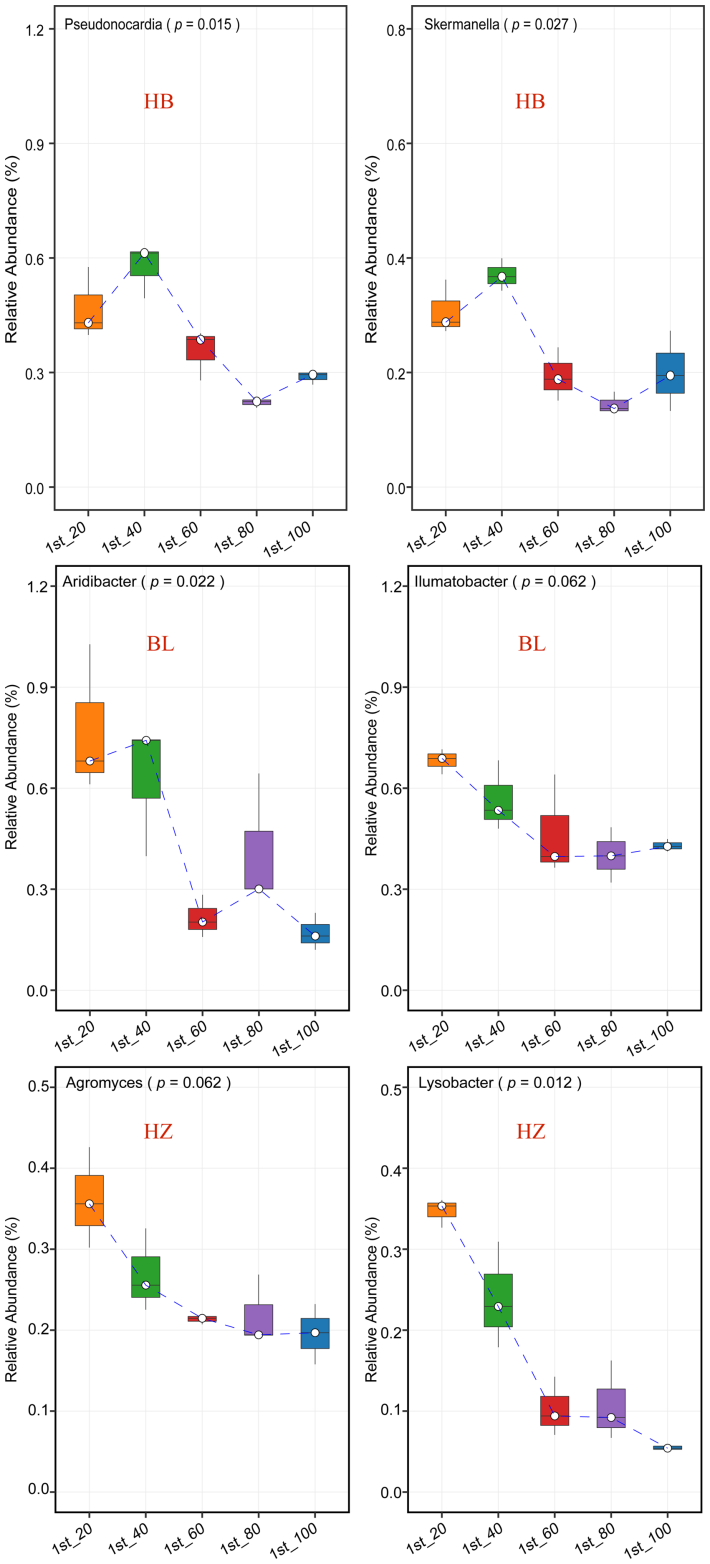
The most connected nodes (i.e., genera) characteristics of the tree networks. The center line of boxplot represents the median, box limits represent upper and lower quartiles and whiskers represent 1.5× interquartile range. HB: the experimental site was Haibei Agricultural and Animal Husbandry Sciences Institute in Qinghai Province, HZ: the experimental site was Gannan Institute of Agricultural Sciences in Gansu Province; BL: the experimental site was Bailang Agricultural Science Institute in Tibet Autonomous Region.

The effects of different soil depths on the relative abundance of nodes in the three microbial co-occurrence networks were investigated (Figure 5 and Supplementary Figure 3). The genera *Pseudonocardia* and *Skermanella* were the hubs of the microbial co-occurrence network of HB. The bacterial genera were enriched at the soil depth of 60 cm (Figure 5, the *p*-values for *Pseudonocardia* and *Skermanella* were 0.015 and 0.027, respectively). On the other hand, the genera *Aridibacter* and *Illumatobacter* were the hubs of the microbial co-occurrence network of BL. The genus *Aridibacter* was enriched at the soil depth of 40 cm (Figure 5, *p-*value = 0.022), but there were no significant changes in the relative abundance of *Illumatobacter* at the five different depths of soil (Figure 5, *p-*value = 0.062). The genera *Agromyces* and *Lysobacter* were the hubs of the microbial co-occurrence network of HZ. In HZ, as the depth of soil increased, the relative abundance of *Lysobacter* decreased significantly (Figure 5, *p-*value = 0.012), but the relative abundance of *Agromyces* did not change significantly (Figure 5, *p-*value = 0.062).

### Relationships between chemical properties of the soil and the rhizosphere microbiota of Qingke plants

**Figure 6:**
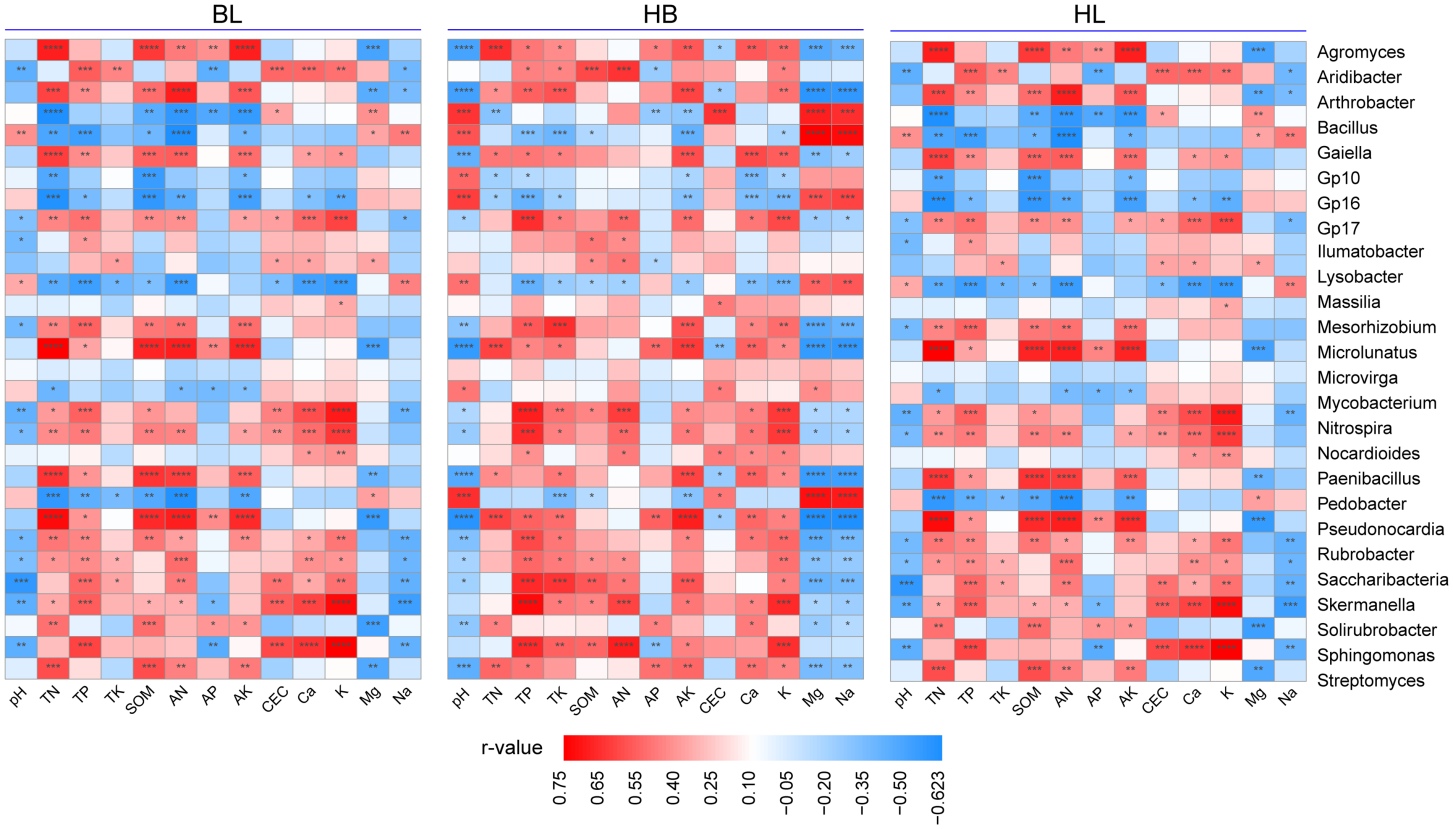
Relationships between fertilization conditions and the rhizosphere microbiota of Qingke plants. Spearman’s correlation analysis to correlate the relative abundance of each of the top 30 genera to the application of each of 13 chemical fertilizers. The genera selected presented in all soil samples with an average relative abundance > 0.1%. HB: the experimental site was Haibei Agricultural and Animal Husbandry Sciences Institute in Qinghai Province, HZ: the experimental site was Gannan Institute of Agricultural Sciences in Gansu Province; BL: the experimental site was Bailang Agricultural Science Institute in Tibet Autonomous Region.

To discover the effects of the soil chemical properties on the rhizosphere microbiota of Qingke plants, we used Spearman’s correlation analysis to correlate the relative abundance of each of the top 30 genera (i.e., the genera were selected with an average relative abundance > 0.1% in all soil samples) to the soil chemical properties (i.e., pH, TN, TP, TK, SOM, AN, AP, AK, CEC, Ca, K, Mg, and Na; Figure 6). As shown in Figure 6, in each of the three experimental sites, the relative abundance of the bacterial genera is strongly correlated with chemical properties of the soil. In BL, TN, TP, SOM, AN, AK, CEC, Ca, and K were positively correlated with the relative abundance of most of the top 30 genera (for example, *Agromyces*, *Arthrobacter*, *Microlunatus*, *Paenibacillus*, and *Pseudonocardia*). On the other hand, the soil chemical properties of Mg, Na, and pH is positively correlated with the relative abundance of a few genera. Compared with BL, HB has a larger number of bacterial genera whose relative abundance is positively correlated with chemical properties of the soil (i.e., TN, TP, SOM, AN, AK, CEC, Ca, and K). In HB, the bacterial genera are *Mycobacterium*, *Nitrospira*, *Nocardioides*, *Paenibacillus*, *Pedobacter*, *Pseudonocardia*, *Rubrobacter*, *Saccharibacteria*, *Skermanella*, *Solirubrobacter*, *Sphingomonas*, and *Streptomyces*. In HB, the application of Mg, Na, and pH was positively correlated with the relative abundance of a few genera. In HZ, the soil chemical properties of TN, TP, SOM, AN, and AK were positively correlated with the relative abundance of *Microlunatus*, *Mycobacterium*, *Nitrospira*, *Paenibacillus*, *Pseudonocardia*, *Skermanella*, and *Sphingomonas*. Moreover, the soil chemical properties of Mg, Na, and pH were positively correlated with the relative abundance of a few genera. Therefore, in all of the three experimental sites, the number of bacterial genera whose relative abundance is positively correlated with the soil chemical properties of Mg, Na, and pH were lower than the number of bacterial genera whose relative abundance is positively correlated with TN, TP, SOM, AN, and AK.

### Effects of geographical locations and fertilization conditions on the growth and yields of Qingke plants

**Figure 7:**
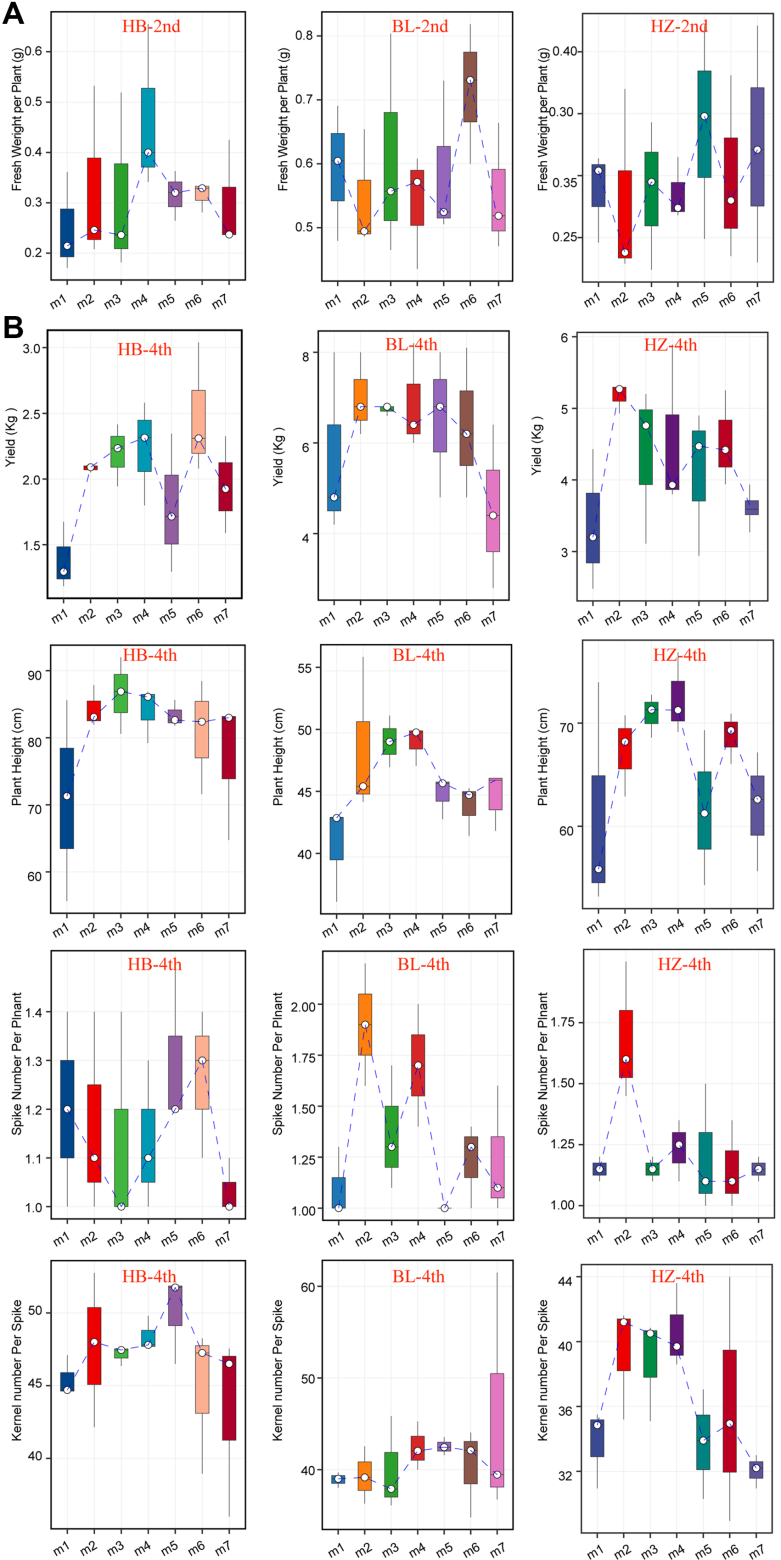
The growth and yields characteristics of Qingke plants with the different fertilization conditions of the three experiments sites. **(A)** The fresh weight per plant of the seeding stage of Qingke plants with the different fertilization conditions at the three experiments sites. **(B)** The plant height, spike number per plant, kernel number per spike, and yields of the mature stage of Qingke plants with the different fertilization conditions of the three experiments sites. The center line of boxplot represents the median, box limits represent upper and lower quartiles and whiskers represent 1.5× interquartile range. 2nd: the seeding stage of Qingke plants. 4th: the mature stage of Qingke plants. m1: Unfertilized; m2: Farmer Practice; m3: 75% Farmer Practice; m4: 75% Farmer Practice +25% Organic manure; m5: 50% Farmer Practice; m6: 50% Farmer Practice +50% Organic manure; m7: 100% Organic manure. HB: The experimental site was Haibei Agricultural and Animal Husbandry Sciences Institute in Qinghai Province, HZ: The experimental site was Gannan Institute of Agricultural Sciences in Gansu Province; BL: the experimental site was Bailang Agricultural Science Institute in Tibet Autonomous Region.

The seven fertilization conditions included in this study affected the growth and yields of Qingke plants, especially when the plants were in the seeding stage (Figure 7A) and mature stage (Figure 7B). The fertilization conditions influenced the heights of Qingke plants, number of spikes in each Qingke plant, number of kernels of each spike, and fresh weight of each Qingke plant (Figure 7). As shown in Figure 7B, fertilization conditions significantly affect the height of Qingke plants. In general, Qingke plants from HB are taller than those from BL and HZ. In addition to fertilization conditions, geographical locations significantly affect the number of spikes in a Qingke plant. In general, the number of spikes in a Qingke plant from BL is higher than the number of spikes in a Qingke plant from HB and that in a Qingke plant from HZ. In addition to the number of spikes in a Qingke plant, the number of kernels in a spike is affected by geographical locations. Overall, the number of kernels in each spike from HB is more than the number of kernels in each spike of a Qingke plant from HZ and that in each spike of a Qingke plant from BL. In each of the three experimental sites, fertilization conditions affect the fresh weight of a Qingke plant (Figure 7A) and yields of Qingke plants (Figure 7B). In all testing sites, the most effective fertilization conditions for improving the yields of Qingke plants are m6 and m5, respectively.

## Discussion

### Effect of different fertilization conditions and geographical locations on the diversity and composition of the rhizosphere microbiota

Studies have reported that the rhizosphere microbiota of grain crops (i.e., wheat and rice) are affected by fertilizers and other interventions affecting the roots of the crops (Kumar and Yadav, 2001; Zhao et al., 2012; Sadet-Bourgeteau et al., 2022). Fertilizers can improve the growth and yields of Qingke plants, and their use in Qingke production has increased rapidly. Studies have shown that fertilizers can alter the structure, diversity, and activity of the soil microbial community (Baker et al., 2009; Wan et al., 2020; Liu et al., 2022). At present, there are limited studies that evaluate the effects of different fertilization conditions and different growth stages of Qingke plants on the rhizosphere microbiota of the plants. In this study, the effects of fertilization conditions, geographical locations, and the growth stages of Qingke plants on the rhizosphere microbiota of the plants were characterized.

Fertilizers can improve the growth and yields of Qingke plants, and their use in Qingke production has increased rapidly. The results of the present study showed that fertilization conditions influenced the composition of the rhizosphere microbiota at the phylum level. In addition to fertilization conditions, geographical locations affected the composition of the rhizosphere microbiota. Actinobacteria, Proteobacteria, and Acidobacteria were the three abundant phyla in the three experimental sites (HB, BL, and HZ). In HB, fertilization conditions significantly changed the relative abundance of Firmicutes, Bacteroidetes, and Gemmatimonadetes. In BL, fertilization conditions significantly altered the relative abundance of Bacteroidetes, Firmicutes, Chloroflexi, and Gemmatimonadetes. Although a study has reported that the application of phosphorus fertilizers increases the relative abundance of Bacteroidetes (Choi et al., 2021; Guan et al., 2022), the effects of the application of other fertilizers on the abundance of the bacterial phylum have not been reported. In general, our results showed the phenomenon that fertilization altered the composition of the rhizosphere microbiota of Qingke in Qingke-producing areas. But the detail changes in rhizosphere microbiota cannot be revealed. Transplant experiment or isolate altered taxa should be carried out to further validate the changes (Jin et al., 2023).

The diversity of soil microbiota is influenced by climatic conditions and geographical locations (Wan et al., 2020; Hinsu et al., 2021; Li et al., 2022). In this study, we also found different geographical locations have the different soil microbiota compositions at phylum and genus levels. Meanwhile, the depths of soil and growth stages of Qingke plants significantly altered the relative abundance of *Nocardioides*, *Sphingomonas*, *Skermanella*, *Bacillus*, *Microvirga*, *Illumatobacter*, *Solirubrobacter*, *Mycobacterium*, *Pseudonocardia*, *Streptomyces*, *Nitrospira*, and *Rubrobacter*. Therefore, the depth of soil and growth stages of Qingke plants are the major drivers of the composition of the rhizosphere microbiota of the plants. To our knowledge, this is the first study that characterizes the effects of fertilization conditions, soil depths, geographical locations, and the growth stages of Qingke plants on the rhizosphere microbiota of the plants.

### Effect of geographical locations on the interaction of the rhizosphere bacteria

Geographical locations, environmental conditions, fertilizer management, and the addition of organic matter are factors that affect the complexity of the soil microbial network (Wei et al., 2015; Gu et al., 2019). In this study, we showed that geographical locations influenced the complexity and stability of the rhizosphere microbiota of Qingke plants. Keystone genera are vital for the rhizosphere microbial community because changes in their abundance will alter the structure and function of the microbial community. Due to their high relative abundance in the three experimental sites, *Pseudonocardia*, *Skermanella*, *Aridibacter*, *Illumatobacter*, *Agromyces*, and *Lysobacter* were the keystone genera of the rhizosphere microbiota of Qingke plants. Moreover, *Pseudonocardia* and *Skermanella* were the hubs of the microbial-occurrence network of HB, *Aridibacter* and *Illumatobacter* were the hubs of the microbial-occurrence network of BL, and *Agromyces* and *Lysobacter* were the hubs of the microbial-occurrence network of HZ.

### Relationships between chemical properties of the soil and the rhizosphere microbiota

This study also demonstrated that fertilization conditions significantly influenced diversity and composition of the rhizosphere microbiota of Qingke plants grown in the three main Qingke-producing areas (Wagg et al., 2018). Chemical properties of soil improve the availability of nutrients to plants, rhizosphere microbiota are excellent alternatives to chemical properties of soil for promoting the growth of Qingke plants (Zeng et al., 2018). Previous studies also have reported that the pH, organic matter (SOM), and microbial biomass carbon (MBC) of soil are important factors that explain differences in the diversity of the fungal community. In this study, we found that the chemical properties of the soil (i.e., TN, TP, SOM, AN, AK, CEC, Ca, and K) was significantly correlated with the relative abundance of the top 30 genera derived from the microbial co-occurrence networks of the three experimental sites. A previous study reported that the pH of soil is a key factor in the composition of the soil fungal community (Mohapatra et al., 2021). In the present study, across different growth stages of Qingke plants, the relative abundance of some genera (i.e., *Aridibacter*, *Bacillus*, and *Gaiella*) was significantly correlated with the pH of soil, implying that the application of chemical fertilizers may lead to soil acidification and changes in the composition of soil microbiota.

### Geographical locations and fertilization conditions influence the growth and yields of Qingke plants

Studies have shown that the application of fertilizers increases crop production, nutrient cycling, and crop resistance to diseases. Chemical fertilizers can improve the yields of Qingke plants. Therefore, their use in different geographical regions of China has increased rapidly (Zeng et al., 2018). However, there are few studies that demonstrate the effects of growth stages of plants on the beneficial effects of fertilizers. In the present study, the effects of fertilization conditions on the growth and yields of Qingke plants in different growth stages were analyzed. Fertilization conditions significantly influenced the growth and yields of Qingke plants, especially the growth and yields of Qingke plants in the seeding and mature stages.

## Data availability statement

The datasets presented in this study can be found in online repositories. The names of the repository/repositories and accession number(s) can be found at: https://www.ncbi.nlm.nih.gov/, PRJNA896263.

## Author contributions

YS, LW, and TY conceived and designed the research. LW and HW performed the experiments and analyzed the data in the research. ML, JX, HB, TC, EY, and YW assisted in the experiments and figure edit. LW wrote the manuscript. YS and TY revised the manuscript. All authors contributed to the article and approved the submitted version.

## Funding

This research was financially supported by the Second Tibetan Plateau Scientific Expedition and Research Program (STEP) (2019QZKK0303), the National Natural Science Foundation of China (General Program 32171958), and the Qinghai Province Natural Science Foundation (2020-ZJ-908).

## Conflict of interest

The authors declare that the research was conducted in the absence of any commercial or financial relationships that could be construed as a potential conflict of interest.

The reviewer YH declared a shared affiliation with the author YW to the handling editor at the time of review.

## Publisher’s note

All claims expressed in this article are solely those of the authors and do not necessarily represent those of their affiliated organizations, or those of the publisher, the editors and the reviewers. Any product that may be evaluated in this article, or claim that may be made by its manufacturer, is not guaranteed or endorsed by the publisher.
