# Corrigendum: Effects of different fertilization conditions and different geographical locations on the diversity and composition of the rhizosphere microbiota of Qingke (*Hordeum vulgare* L.) plants in different growth stages

**DOI:** 10.3389/fmicb.2023.1268063

**Published:** 2023-08-08

**Authors:** Lei Wang, Handong Wang, Meijin Liu, Jinqing Xu, Haiyan Bian, Tongrui Chen, En You, Chao Deng, Youhai Wei, Tianyu Yang, Yuhu Shen

**Affiliations:** ^1^Laboratory for Research and Utilization of Qinghai Tibetan Plateau Germplasm Resources, Northwest Institute of Plateau Biology, Chinese Academy of Sciences, Xining, China; ^2^Key Laboratory of Adaptation and Evolution of Plateau Biota, Northwest Institute of Plateau Biology, Chinese Academy of Sciences, Xining, China; ^3^Qinghai Provincial Key Laboratory of Crop Molecular Breeding, Northwest Institute of Plateau Biology, Chinese Academy of Sciences, Xining, China; ^4^Gannan Institute of Agricultural Sciences, Hezuo, China; ^5^University of Chinese Academy of Sciences, Beijing, China; ^6^Academy of Agriculture and Forestry Science, Qinghai University, Xining, China; ^7^Crop Research Institute, Gansu Academy of Agricultural Sciences, Lanzhou, China; ^8^Innovation Academy for Seed Design, Chinese Academy of Sciences, Xining, China

**Keywords:** Qingke, rhizosphere microbiota, fertilization condition, geographical location, diversity

In the published article, there was an error in the Funding statement. The funding statement for the Qinghai Province Natural Science Foundation was displayed as “2022-ZJ-908”.

The corrected Funding statement appears below.

