# Supplementary figures and images for: Effects of different fertilization conditions and different geographical locations on the diversity and composition of the rhizosphere microbiota of Qingke (Hordeum vulgare L.) plants in different growth stages
Source: Front Microbiol. 2023 May 4;14:1094034. doi: 10.3389/fmicb.2023.1094034 (PMC10192736; doi:10.3389/fmicb.2023.1094034)

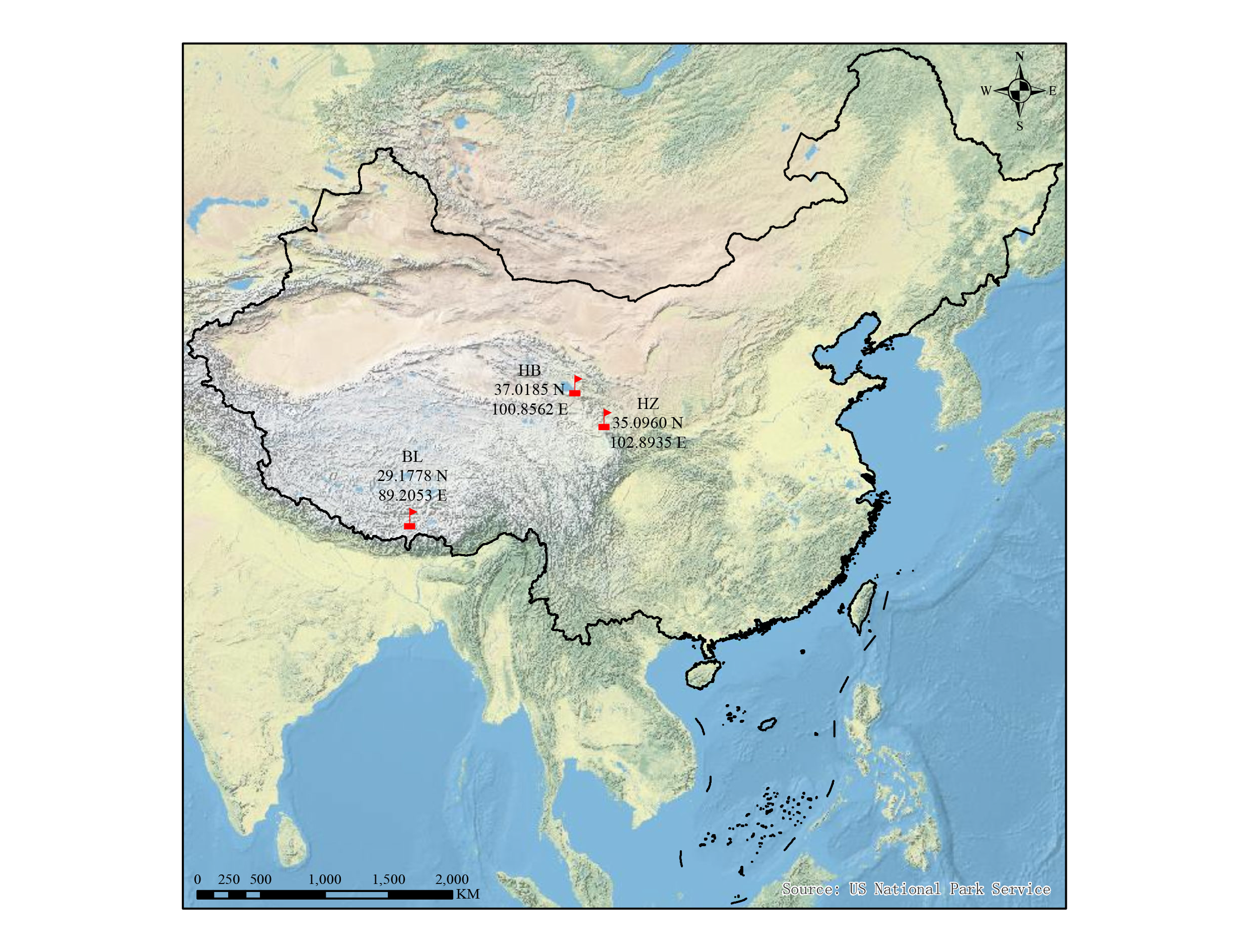

Supplement: Supplementary file 1 [file Data_Sheet_1.ZIP › new_supplementary0406/New_Supp_Fig_1.tif]

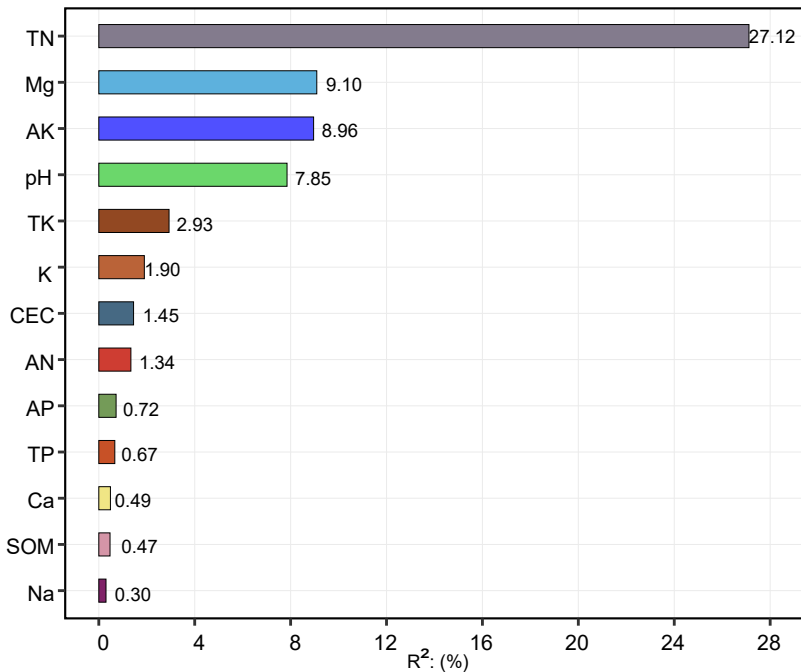

Supplement: Supplementary file 1 [file Data_Sheet_1.ZIP › new_supplementary0406/New_Supp_Fig_2.pdf]

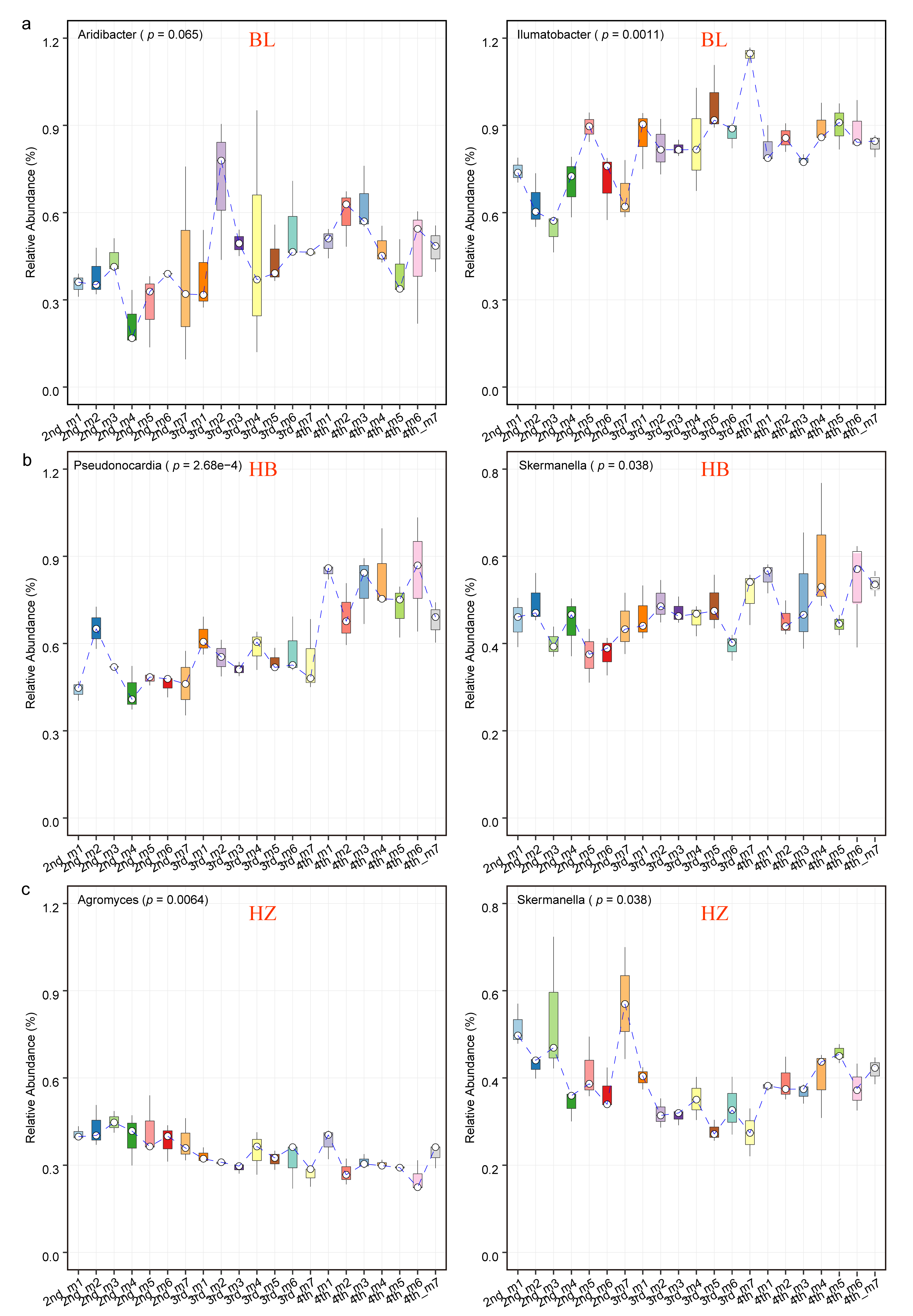

Supplement: Supplementary file 1 [file Data_Sheet_1.ZIP › new_supplementary0406/New_Supp_Fig_3.tif]
